# Gender-specific differences in risk for intimate partner violence in South Korea

**DOI:** 10.1186/1471-2458-14-415

**Published:** 2014-05-01

**Authors:** Minjee Lee, Katherine M Stefani, Eun-Cheol Park

**Affiliations:** 1 Department of Health Management and Policy, College of Public Health, University of Iowa, 105 River Street, N265 CPHB, 52242 Iowa City, IA, USA; 2 Department of Research Affairs, Yonsei University College of Medicine, 50 Yonsei-ro, Seodaemun-gu, 120-752 Seoul, South Korea; 3 Department of Preventive Medicine and Public Health, Institute of Health Services Research, Yonsei University College of Medicine, 134 Shinchon-dong, Seodaemun-gu, 120-752 Seoul, South Korea

**Keywords:** Gender, Intimate partner violence, South Korea

## Abstract

**Background:**

Various risk factors of intimate partner violence (IPV) have been found to vary by gender. South Korea has one of the highest prevalences of IPV in the world; however, little is known about potential risk factors of IPV and whether gender influences this relationship.

**Methods:**

Using data from the 2006 Korea Welfare Panel Study, 8,877 married participants (4,545 men and 4,332 women) aged ≥30 years were included. Reported IPV was categorized as verbal or physical IPV and the association between IPV and related factors was assessed by multivariate logistic regression analysis.

**Results:**

Women were significantly more likely than men were to report IPV victimization (verbal 28.2% vs. 24.4%; physical 6.9% vs. 3.4%). Wor odds of physical perpetration than women satisfied with their family. Moreover, alcohol intake was significantly associated with IPV perpetration and victimization in both genders.

**Conclusion:**

Significant gender-specific differences were found among factors related to perpetrating violence and being a victim of violence among adults in heterosexual relationships in South Korea.

## Background

Intimate partner violence (IPV) is a worldwide public health problem as well as a serious social problem in South Korea [1-3]. IPV is characterized as any behavior within an intimate relationship that causes physical, psychological, or sexual harm of one partner to another [1]. According to the 2010 national survey in South Korea, the prevalence of reported IPV was 53.8%, and 81.9% of this violence was perpetrated by husbands against their wives [4].

IPV causes a wide range of negative effects on the health of women [5-7] and children, such as injury, chronic pain, gastrointestinal problems, sexually transmitted diseases, depression, and post-traumatic stress disorder [8-10]. In addition, a significant number of deaths among women are considered to result from IPV [11,12]. Moreover, children who witness IPV in their home are also significantly more likely to experience or perpetrate IPV than are children who do not [13].

The prevalence of reported partner violence varies greatly (15%-71%) among various countries [1,14,15]. In South Korea, the special law to Prevent Family Violence and Protect the Victim mandates that Korean nationals be surveyed triennially. According to this survey, the prevalence of reported IPV dropped from 53.8% in 2010 to 45.5% in 2013 [16].

IPV typically results due to gender inequality and is frequently considered a form of gender-based violence [1]. Violence against women has been the focus of most research, yet little is known about the prevalence of violence perpetrated against men who are in a heterosexual relationship [17,18]. Some studies have found the prevalence of violence against men to be equivalent to that against women [18,19]. Similarly, in South Korea, previous research has highlighted the scope and risk factors of violence against women; however, few studies have investigated the prevalence of violence against men or the possible risk factors associated with male perpetration and victimization [20,21].

Factors associated with IPV victimization among women include pregnancy, depressive symptoms, smoking, alcohol consumption, low socioeconomic status, experiencing IPV during childhood, and witnessing IPV perpetration against their mother [22-28]. Tumwesigye and colleagues [27] investigated IPV victimization among women in Uganda and found socioeconomic status including education, employment status, income, and education level as well as the employment status of the partner to be potential risk factors. Furthermore, Lemon and colleagues [28] reported that current smoking and frequent alcohol use are related to IPV victimization among women living in the US (Rhode Island). Although few studies have examined IPV victimization among men, alcohol consumption, low socioeconomic status, experiencing IPV during childhood, and witnessing IPV perpetration against their mother were factors associated with IPV perpetration [29-33]. In addition, a study that compared both men and women found alcohol dependency to be associated with severe physical IPV perpetration in the New Zealand Birth Cohort [33].

In addition, lacking a social network, emotional support, and having low perceived life satisfaction were found to be related with IPV among men and women [2,33-37]. For example, South Korean women with poor social/support networks were more likely to experience IPV victimization [2,35,36] as well as continued abuse [37]. Among Chinese men and women, life dissatisfaction was related with IPV victimization [34]. Furthermore, a weak social support system was strongly associated with physical IPV victimization among women in New Zealand [33].

Although the aforementioned studies identified some factors associated with IPV, the limited sample size, lack of gender-specific analyses, and a lack of consideration for IPV perpetration among women and IPV victimization among men warrant further study. Furthermore, previous research has not considered factors related to each type of IPV (verbal and physical). To this end, we investigated whether gender-specific differences exist in the prevalence of IPV as well as the type of the violence that was perpetrated or experienced.

## Methods

### Data and sample

Data from the nationally representative 2006 Korea Welfare Panel Study (KOWEPS) performed by the Korean Institute of Social and Health Affairs in conjunction with Social Welfare Research Institute of Seoul National University were used for this study. Details of this study have been published elsewhere [38,39]. Briefly, KOWEPS is a comprehensive dataset that provides a variety of information on families and individuals with respect to their social service needs, heath care utilization patterns, economic and demographic background, sources of income, and subjective emotional and behavioral health status. A stratified, multistage, probability design was used. Men and women older than 19 were selected from sampling units using household registries. In total, 7,072 households participated in the survey. Trained interviewers conducted all surveys at participants’ homes, and all participants provided informed consent before participating in the survey.

In the 2006 dataset, 18,856 men and women were recruited. Of them, 16,084 (85.29%) men and women aged 19 or older participated in the survey. For our analyses, only those who were older than 30 years old and married were included leaving 9,667 men and women. The 16 participants who were younger than 30 and married were excluded from our analysis because, according to the National Statistical Office in South Korea, the average age of marriage among males and females was 32.1 was and 29.4 in 2012, respectively [40]. By limiting our analysis to those 30 and older, we hoped to include a more representative population of married couples. After exclusion for those missing any relevant data (n = 790), a total of 8,877 participants (4,545 men and 4,332 women) who reported being married at the time of the survey were included in the analysis.

### Measurement of IPV

During data collection, participants were asked 13 questions pertaining to the level and type of violence experienced in their marriage over the past 12 months. Verbal IPV was assessed by asking how often in the past 12 months their spouse was (1) insulting, (2) made a malicious remark, or (3) threatened them. Physical IPV was assessed across ten violent activities. Respondents were asked how often in the past 12 months their spouse perpetrated the following physically violent activities at him/her: (1) threw something, (2) pushed, (3) slapped, (4) kicked or punched, (5) used an object to hit, (6) beat, (7) threatened using a weapon like a knife, (8) choked, (9) caused a sprain or bruise, or (10) caused him/her to be hospitalized after a violent encounter. These questions were adopted from the Conflict Tactics Scale [39]. In addition, participants were also asked how often they perpetrated any of these acts against their spouse, and their answers were recorded as never, 1–2 times, 3–5 times, 6–10 times, or >10 times throughout the previous 12 months. A person was considered to have experienced or perpetrated IPV if a violent event occurred once or more over the past 12 months, and this was recorded as the binary outcome variable (yes or no) in our analysis.

### Independent variables

The statistical models used in this study were created based on variables reported in previous studies [15,41]. Nine variables from three domains (related to socio-demographic factors, family/life satisfaction, and health behaviors) that have been robustly linked to IPV in epidemiological studies for IPV victimization among women and IPV perpetration among men were selected [27,28,42]. The socio-demographic factors include age, education, household income, employment status, and the perceived wealth during childhood. Participants’ subjective levels of satisfaction in either family relationships or one’s personal life was also measured along with health behaviors such as smoking and alcohol intake [27,28,42].

Participants were divided into five age groups, 30–39, 40–49, 50–59, 60–69, and ≥70 years, for the analysis. In addition, education level was stratified into three groups based on the highest level of education achieved as elementary school, middle or high school, or university or higher. Income was calculated according to the equivalized household income equation as the sum of the total household income from all sources including earned income, income from assets, and miscellaneous income divided by the square root of the number of household members. The equivalized income was then divided into quartiles [39]. Employment status was categorized as either being employed full time, part time, being self-employed, or unemployed. Participants’ perceived level of wealth during childhood was categorized as either poor, average or wealthy. The perceived level of satisfaction with family and life was recorded as not satisfied, neutral, or satisfied. Smoking was categorized as never or ever. Moreover, participants were divided into four groups based on the average number of drinks consumed at one time as a nondrinker, light drinker (1–4 drinks), moderate drinker (5–9 drinks), or heavy drinker (>10 drinks).

### Statistical analysis

Descriptive statistics were used to describe participant characteristics and report the number and percentage of participants for each variable. In addition, the prevalence of IPV was calculated for all variables. Odds ratios (OR) with 95% confidence intervals (CI) were calculated to measure the strength of the association between IPV and all possible IPV-related factors in this study population. Multivariate logistic regression models, with IPV as the dependent variable, were used to calculate gender-specific ORs. Fully adjusted ORs were calculated after controlling for all potential confounders (age, education, household income, employment status, perceived wealth during childhood, satisfaction with family and life, smoking, and alcohol intake). Sampling weights were also added to aid in generalizing our findings to the entire population of South Korea. All statistical analyses were performed using SAS version 9.2 (SAS Inc., Cary, NC, USA), and a *p*-value >0.05 was considered statistically significant.

## Results

Gender-specific data on the characteristics of the study population and the prevalence of IPV are shown in Tables 1 and 2, respectively.

**Table 1:** Descriptive data across the type of intimate partner violence among men

**Variables**	**Total**	**%**	**Victim**						**Perpetrator**					
			**Verbal**	**%**	**p-value**	**Physical**	**%**	**p-value**	**Verbal**	**%**	**p-value**	**Physical**	**%**	**p-value**
**Age (years)**														
30-39	401	8.82	104	25.9	<.0001	26	6.5	<.0001	105	26.2	<.0001	37	9.2	<.0001
40-49	1103	24.27	322	29.2		69	6.3		317	28.7		77	7.0	
50-59	955	21.01	254	26.6		35	3.7		267	28.0		54	5.7	
60-69	750	16.5	159	21.2		12	1.6		176	23.5		28	3.7	
70+	1336	29.39	269	20.1		13	1.0		286	21.4		35	2.6	
**Education**														
None or elementary school	1056	23.23	253	24.0	0.936	23	2.2	0.021	278	26.3	0.645	38	3.6	0.034
Middle or high school	2143	47.15	525	24.5		75	3.5		541	25.2		123	5.7	
University or higher	1346	29.61	330	24.5		57	4.2		332	24.7		70	5.2	
**Household income**														
Quartile 1	1086	23.89	233	21.5	0.058	25	2.3	0.080	263	24.2	0.777	40	3.7	0.032
Quartile 2	1106	24.33	276	25.0		46	4.2		280	25.3		53	4.8	
Quartile 3	1162	25.57	305	26.2		45	3.9		303	26.1		74	6.4	
Quartile 4	1191	26.2	294	24.7		39	3.3		305	25.6		64	5.4	
**Employment status**														
Employed full time	2161	47.55	521	24.1	0.006	86	4.0	0.022	523	24.2	0.015	107	5.0	<.0001
Employed part time	989	21.76	276	27.9		36	3.6		288	29.1		77	7.8	
Self employed	491	10.8	121	24.6		17	3.5		127	25.9		17	3.5	
Unemployed	904	19.89	190	21.0		16	1.8		213	23.6		30	3.3	
**Perceived wealth in childhood**														
Poor	2146	47.22	520	24.2	0.843	67	3.1	0.319	559	26.0	0.431	110	5.1	0.920
Average	1878	41.32	465	24.8		73	3.9		470	25.0		93	5.0	
Wealthy	521	11.46	123	23.6		15	2.9		122	23.4		28	5.4	
**Satisfaction with family relationships**														
Satisfied	3682	81.01	814	22.1	<.0001	107	2.9	<.0001	839	22.8	<.0001	148	4.0	<.0001
Neutral	732	16.11	238	32.5		30	4.1		253	34.6		62	8.5	
Not satisfied	131	2.88	56	42.7		18	13.7		59	45.0		21	16.0	
**Satisfaction with one’s personal life**														
Satisfied	1668	36.7	332	19.9	<.0001	37	2.2	<.0001	339	20.3	<.0001	55	3.3	<.0001
Neutral	2167	47.68	547	25.2		71	3.3		575	26.5		114	5.3	
Not satisfied	710	15.62	229	32.3		47	6.6		237	33.4		62	8.7	
**Smoking**														
Never	2351	51.73	512	21.8	<.0001	57	2.4	0.000	525	22.3	<.0001	91	3.9	0.000
Ever	2194	48.27	596	27.2		98	4.5		626	28.5		140	6.4	
**Alcohol intake**														
Nondrinker	1373	30.21	243	17.7	<.0001	25	1.8	<.0001	253	18.4	<.0001	42	3.1	<.0001
Light drinker (1-4 drinks)	1053	23.17	249	23.6		28	2.7		242	23.0		38	3.6	
Moderate drinker (5-9 drinks)	1381	30.39	380	27.5		53	3.8		422	30.6		77	5.6	
Heavy drinker (10 ≤ drinks)	738	16.24	236	32.0		49	6.6		234	31.7		74	10.0	
**Total**	4545	100	1108	24.4		155	3.4		1151	25.3		231	5.1	

**Table 2:** Descriptive data across the type of intimate partner violence among Women

**Variables**	**Total**	**%**	**Victim**						**Perpetrator**					
			**verbal**	**%**	**p-value**	**physical**	**%**	**p-value**	**verbal**	**%**	**p-value**	**physical**	**%**	**p-value**
**Age (years)**														
30-39	658	15.19	176	26.7	0.001	65	9.9	<.0001	197	29.9	<.0001	42	6.4	<.0001
40-49	1175	27.12	372	31.7		96	8.2		363	30.9		61	5.2	
50-59	877	20.24	269	30.7		71	8.1		244	27.8		25	2.9	
60-69	762	17.59	194	25.5		39	5.1		174	22.8		11	1.4	
70+	860	19.85	211	24.5		27	3.1		180	20.9		9	1.0	
**Education**														
None or elementary school	1400	32.32	404	28.9	0.448	78	5.6	0.061	358	25.6	0.185	21	1.5	<.0001
Middle or high school	2068	47.74	589	28.5		157	7.6		549	26.5		79	3.8	
University or higher	864	19.94	229	26.5		63	7.3		251	29.1		48	5.6	
**Household income**														
Quartile 1	1038	23.96	300	28.9	0.766	59	5.7	0.239	273	26.3	0.932	22	2.1	0.027
Quartile 2	1055	24.35	306	29.0		74	7.0		288	27.3		36	3.4	
Quartile 3	1098	25.35	304	27.7		87	7.9		297	27.0		39	3.6	
Quartile 4	1141	26.34	312	27.3		78	6.8		300	26.3		51	4.5	
**Employment status**														
Employed full time	2094	48.34	582	27.8	0.072	161	7.7	<.0001	591	28.2	0.000	97	4.6	<.0001
Employed part time	875	20.2	277	31.7		75	8.6		263	30.1		28	3.2	
Self employed	189	4.36	50	26.5		15	7.9		44	23.3		10	5.3	
Unemployed	1174	27.1	313	26.7		47	4.0		260	22.1		13	1.1	
**Perceived wealth in childhood**														
Poor	1548	35.73	463	29.9	0.179	118	7.6	0.316	440	28.4	0.050	47	3.0	0.052
Average	2219	51.22	605	27.3		146	6.6		587	26.5		89	4.0	
Wealthy	565	13.04	154	27.3		34	6.0		131	23.2		12	2.1	
**Satisfaction with family relationships**														
Satisfied	3396	78.39	841	24.8	<.0001	168	4.9	<.0001	811	23.9	<.0001	88	2.6	<.0001
Neutral	780	18.01	294	37.7		88	11.3		264	33.8		34	4.4	
Not satisfied	156	3.6	87	55.8		42	26.9		83	53.2		26	16.7	
**Satisfaction with one’s personal life**														
Satisfied	1451	33.49	303	20.9	<.0001	58	4.0	<.0001	296	20.4	<.0001	37	2.5	0.000
Neutral	2216	51.15	654	29.5		154	6.9		596	26.9		71	3.2	
Not satisfied	665	15.35	265	39.8		86	12.9		266	40.0		40	6.0	
**Smoking**														
Never	4254	98.2	1199	28.2	0.800	292	6.9	0.775	1134	26.7	0.416	141	3.3	0.006
Ever	78	1.8	23	29.5		6	7.7		24	30.8		7	9.0	
**Alcohol intake**														
Nondrinker	3026	69.85	778	25.7	<.0001	179	5.9	0.000	721	23.8	<.0001	81	2.7	<.0001
Light drinker (1-4 drinks)	1046	24.15	345	33.0		88	8.4		342	32.7		44	4.2	
Moderate drinker (5-9 drinks)	213	4.92	81	38.0		24	11.3		75	35.2		17	8.0	
Heavy drinker (10 ≤ drinks)	47	1.08	18	38.3		7	14.9		20	42.6		6	12.8	
**Total**	4332	100	1222	28.2		298	6.9		1158	26.7		148	3.4	

The majority of men reported working full time (47.6%), had at least a middle or high school education (47.2%), were non-smokers (51.7%), and moderate drinkers (30.4%). For men, the prevalence of verbal IPV was 24.4% for victimization and 25.3% for perpetration. The prevalence of physical IPV was 3.4% for victimization and 5.1% for perpetration (Table 1).

The majority of women in this study population were employed full time (48.3%) and had at least a middle or high school education (47.7%). In addition, most women were nonsmokers (98.2%) and nondrinkers (69.9%). For women, the prevalence of verbal IPV was 28.2% for victimization and 26.7% for perpetration. The prevalence of physical IPV was 6.9% for victimization and 3.4% for perpetration (Table 2). For both men and women, the prevalence of victimization and perpetration may be overlapped, thus should not be considered independent measures.

Women were significantly more likely than men were to report being a victim of IPV (verbal: 28.2% vs. 24.4% [*p* < .0001], physical: 6.9% vs. 3.4% [*p* < .0001]). In addition, women tended to perpetrate verbal violence against their spouse more often than men were (26.7% vs. 25.3%). However, 5.1% of men and 3.4% of women reported perpetrating physical violence against their spouse. A similar proportion of men and women reported male-to-female violence, yet more women than men reported female-to-male violence.

Among males, household income, perceived wealth during childhood, and smoking were not significantly associated with IPV victimization nor with IPV perpetration. Men with a middle or high school education were significantly less likely to report perpetrating verbal IPV than were men with only an elementary school education. In addition, men employed part time were significantly more likely to perpetrate physical IPV than men employed full time were. Moreover, having a high level of satisfaction with family relationships and one’s personal life made men less likely to perpetrate both verbal and physical IPV. Compared with men who were satisfied with their family relationships, the adjusted OR for men dissatisfied with their family relationships was 2.44 (95% CI: 1.66-3.58) for verbal IPV victimization, 5.49 (95% CI: 2.91-10.37) for physical IPV victimization, 2.54 (95% CI: 1.74-3.71) for verbal IPV perpetration, and 4.68 (95% CI: 2.66-8.27) for physical IPV perpetration. However, the adjusted OR among men who were dissatisfied with their personal life was 1.76 (95% CI: 1.39-2.22) for verbal IPV victimization, 3.07 (95% CI: 1.79-5.26) for physical IPV victimization, 1.69 (95% CI: 1.34-2.13) for verbal IPV perpetration, and 2.51 (95% CI: 1.60-3.95) for physical IPV perpetration when compared to men who were satisfied with their personal life. Among the measured health behaviors, men reporting high alcohol intake were more likely to have experienced verbal IPV victimization, physical IPV victimization, verbal IPV perpetration, and physical IPV perpetration. Compared with non-drinkers, the adjusted OR of heavy drinkers was 2.06 (95% CI: 1.65-2.58) for verbal IPV victimization, 2.54 (95% CI: 1.49-4.31) for physical IPV victimization, 2.00 (95% CI: 1.60-2.50) for verbal IPV perpetration, and 2.81 (95% CI: 1.84-4.30) for physical IPV perpetration (Table 3).

**Table 3:** Factors associated with intimate partner violence reported by men

**Variables**	**Victimization**				**Perpetration**			
	**Verbal**		**Physical**		**Verbal**		**Physical**	
**Age (years)**								
30-39	1.00		1.00		1.00		1.00	
40-49	1.16	(0.89 - 1.51)	0.92	(0.57 - 1.50)	1.11	(0.85 - 1.45)	0.70	(0.45 - 1.07)
50-59	0.98	(0.74 - 1.30)	0.46	(0.26 - 0.81)	1.02	(0.77 - 1.35)	0.52	(0.32 - 0.83)
60-69	0.75	(0.55 - 1.03)	0.18	(0.08 - 0.39)	0.81	(0.60 - 1.11)	0.36	(0.20 - 0.64)
70+	0.74	(0.54 - 1.02)	0.08	(0.04 - 0.20)	0.73	(0.53 - 1.00)	0.27	(0.15 - 0.51)
**Education**								
None or elementary school	1.00		1.00		1.00		1.00	
Middle or high school	0.81	(0.66 - 1.01)	0.69	(0.38 - 1.26)	0.81	(0.66 - 1.00)	1.09	(0.69 - 1.71)
University or higher	0.82	(0.62 - 1.07)	0.74	(0.37 - 1.50)	0.83	(0.63 - 1.08)	0.97	(0.56 - 1.70)
**Employment status**								
Employed full time	1.00		1.00		1.00		1.00	
Employed part time	1.13	(0.94 - 1.36)	0.73	(0.48 - 1.13)	1.18	(0.98 - 1.42)	1.45	(1.05 - 2.02)
Self employed	1.13	(0.88 - 1.45)	1.22	(0.68 - 2.19)	1.16	(0.90 - 1.48)	0.83	(0.47 - 1.44)
Unemployed	0.93	(0.74 - 1.17)	0.82	(0.44 - 1.54)	1.02	(0.82 - 1.28)	0.99	(0.61 - 1.60)
**Satisfaction with family relationships**								
Satisfied	1.00		1.00		1.00		1.00	
Neutral	1.68	(1.39 - 2.02)	1.58	(1.01 - 2.47)	1.73	(1.43 - 2.08)	2.38	(1.70 - 3.35)
Not satisfied	2.44	(1.66 - 3.58)	5.49	(2.91 - 10.37)	2.54	(1.74 - 3.71)	4.68	(2.66 - 8.27)
**Satisfaction with one’s personal life**								
Satisfied	1.00		1.00		1.00		1.00	
Neutral	1.30	(1.10 - 1.54)	1.62	(1.05 - 2.51)	1.31	(1.11 - 1.55)	1.55	(1.08 - 2.21)
Not satisfied	1.76	(1.39 - 2.22)	3.07	(1.79 - 5.26)	1.69	(1.34 - 2.13)	2.51	(1.60 - 3.95)
**Alcohol intake**								
Nondrinker	1.00		1.00		1.00		1.00	
Light drinker (1-4 drinks)	1.50	(1.22 - 1.83)	1.46	(0.84 - 2.57)	1.36	(1.11 - 1.67)	1.17	(0.74 - 1.84)
Moderate drinker (5-9 drinks)	1.71	(1.40 - 2.08)	1.62	(0.97 - 2.70)	1.92	(1.58 - 2.33)	1.58	(1.05 - 2.38)
Heavy drinker (10 ≤ drinks)	2.06	(1.65 - 2.58)	2.54	(1.49 - 4.31)	2.00	(1.60 - 2.50)	2.81	(1.84 - 4.30)

Results are presented as adjusted odds ratios and (95% confidence intervals), and adjusted for age, education, household income, employment status, perceived wealth during childhood, satisfaction with family relationships, satisfaction with one’s personal life, smoking, and alcohol intake.

Among women, age was significantly associated with all types of IPV, but household income, employment status, perceived wealth during childhood, and smoking were not. In addition, women with at least a middle or high school education were significantly less likely to report verbal IPV victimization and physical IPV perpetration than women with less education were. Similar to men, increased alcohol intake was associated with an increased odds of verbal and physical IPV perpetration. However, unlike men, alcohol drinking was not significantly associated with physical IPV victimization. Compared with non-drinkers, the adjusted ORs of moderate drinkers for verbal IPV victimization, verbal IPV perpetration, and physical IPV perpetration were 1.68 (95% CI: 1.24-2.27), 1.53 (95% CI: 1.13-2.08), and 2.15 (95% CI: 1.21-3.83), respectively. Moreover, women with a high level of family and life satisfaction were less likely to report verbal IPV victimization, physical IPV victimization, verbal IPV perpetration, and physical IPV perpetration; these findings are similar to that among men. Compared with women who were satisfied with their family relationships, the adjusted ORs among women dissatisfied with their relationships were 3.17 (95% CI: 2.23-4.52) for verbal IPV victimization, 6.68 (95% CI: 4.23-10.56) for physical IPV victimization, 2.78 (95% CI: 1.95-3.96) for verbal IPV perpetration, and 9.46 (95% CI: 5.21-17.19) for physical IPV perpetration. Moreover, the adjusted ORs among women dissatisfied with their personal life was 1.96 (95% CI: 1.55-2.49) for verbal IPV victimization, 2.52 (95% CI: 1.64-3.89) for physical IPV victimization, 2.36 (95% CI: 1.85-3.00) for verbal IPV perpetration, and 2.23 (95% CI: 1.22-4.08) for physical IPV perpetration when compared to women who reported being satisfied with their personal life (Table 4).

**Table 4:** Factors associated with intimate partner violence reported by women

**Variables**	**Victimization**				**Perpetration**			
	**Verbal**		**Physical**		**Verbal**		**Physical**	
**Age (years)**								
30-39	1.00		1.00		1.00		1.00	
40-49	1.23	(0.99 - 1.54)	0.75	(0.53 - 1.07)	1.04	(0.84 - 1.30)	0.79	(0.52 - 1.22)
50-59	1.06	(0.82 - 1.36)	0.63	(0.42 - 0.96)	0.86	(0.66 - 1.10)	0.43	(0.24 - 0.77)
60-69	0.69	(0.51 - 0.93)	0.31	(0.18 - 0.52)	0.57	(0.42 - 0.78)	0.22	(0.09 - 0.50)
70+	0.53	(0.38 - 0.75)	0.15	(0.08 - 0.28)	0.43	(0.30 - 0.61)	0.14	(0.05 - 0.39)
**Education**								
None or elementary school	1.00		1.00		1.00		1.00	
Middle or high school	0.77	(0.61 - 0.96)	0.70	(0.46 - 1.07)	0.73	(0.57 - 0.92)	1.07	(0.53 - 2.17)
University or higher	0.76	(0.56 - 1.02)	0.67	(0.39 - 1.13)	0.87	(0.64 - 1.17)	1.38	(0.62 - 3.11)
**Satisfaction with family relationships**								
Satisfied	1.00		1.00		1.00		1.00	
Neutral	1.66	(1.39 - 1.99)	2.43	(1.80 - 3.28)	1.48	(1.23 - 1.78)	1.94	(1.24 - 3.04)
Not satisfied	3.17	(2.23 - 4.52)	6.68	(4.23 - 10.56)	2.78	(1.95 - 3.96)	9.46	(5.21 - 17.19)
**Satisfaction with one’s personal life**								
Satisfied	1.00		1.00		1.00		1.00	
Neutral	1.48	(1.25 - 1.76)	1.63	(1.16 - 2.29)	1.45	(1.22 - 1.73)	1.45	(0.92 - 2.27)
Not satisfied	1.96	(1.55 - 2.49)	2.52	(1.64 - 3.89)	2.36	(1.85 - 3.00)	2.23	(1.22 - 4.08)
**Alcohol intake**								
Nondrinker	1.00		1.00		1.00		1.00	
Light drinker (1-4 drinks)	1.38	(1.17 - 1.62)	1.21	(0.91 - 1.61)	1.45	(1.23 - 1.70)	1.13	(0.76 - 1.67)
Moderate drinker (5-9 drinks)	1.68	(1.24 - 2.27)	1.58	(0.98 - 2.53)	1.53	(1.13 - 2.08)	2.15	(1.21 - 3.83)
Heavy drinker (10 ≤ drinks)	1.47	(0.80 - 2.70)	1.78	(0.75 - 4.18)	1.83	(1.00 - 3.35)	3.18	(1.23 - 8.20)

Results are presented as adjusted odds ratios and (95% confidence intervals), and adjusted for age, education, household income, employment status, perceived wealth during childhood, satisfaction with family relationships, satisfaction with one’s personal life, smoking, and alcohol intake.

## Discussion

In this nationally representative population of Korean men and women, significant gender-specific differences were evident for the prevalence of IPV and its associated factors. Factors significantly associated with an increased likelihood of IPV victimization and perpetration among men and women were the subjective measures of family and personal life satisfaction as well as alcohol intake. In addition, the prevalence of IPV victimization was significantly higher among women than among men.

Although gender-specific data on IPV are limited, our findings are consistent with estimates gathered from previous studies with smaller samples of South Korean adults and population-based studies conducted in other Asian countries [20,42-45], which found higher exposures to IPV among women than men.

Investigation into the physical perpetration of IPV revealed that males had slightly higher rates of perpetration than that of females (males, 5.1% vs. females, 3.4%). In addition, women reported a lower rate of experiencing male perpetration than that of experiencing victimization. However, the rate of female perpetration was roughly aligned with the rate of victimization that was reported by men. One interpretation for this discrepancy may be that men underreported IPV perpetration due to a social desirability bias. Similar disparities in reporting have been found in studies conducted in other countries [46].

The majority of research surrounding IPV has addressed violence against women, examining prevalence estimates of female victimization and male perpetration. In the current study, rates of verbal IPV victimization among females (28.2%) fell within the range of previous estimates (24.6%-55.0%) [47,48], although male rates of verbal perpetration (25.3%) were slightly lower than previous estimates (31.8%-42.3%) [49,50]. However, limitations between survey methods make comparing our results with previous studies difficult; inconsistencies in defining violence and variations in survey periods may have created discrepancies between these estimates.

Among men, low education level was positively associated with perpetrating verbal violence; however, among women, low education level was positively associated with both victimizing and perpetrating verbal IPV. Previous studies have reported similar associations and have strongly suggested that future violence prevention programs should aim to increase levels of education and understanding about IPV [47].

Our results also confirm the finding of other studies that IPV victimization and perpetration are likely to occur regardless of one’s drinking habits [46,47]. However, we cannot determine whether alcohol is a risk factor of or a result of IPV due to the cross-sectional design of our study. Nevertheless, our findings support previous assertions that alcohol interventions may be a crucial component of future violence prevention programs [19,51,52].

Our study has important limitations. First, the prevalence of IPV was measured over the previous 12 months; therefore, these data may have underestimated the actual prevalence of IPV in this study population. Second, the KOWEPS dataset did not measure experiences with IPV during childhood such as any exposures to domestic violence or witnessing IPV perpetration among their parents. Third, the KOWEPS dataset did not ask participants who were married whether they live with their spouse. We cannot assume that all married couples live with their spouse and this factor may influence the prevalence of IPV; therefore, future studies should investigate whether this factor is associated with IPV. Fourth, IPV related to sexual abuse was not included in the KOWEPS survey. Last, the confidence intervals estimated in our analyses were wide; therefore, future prospective studies with large sample sizes are needed to better understand these relationships.

In this nationally representative study, we found that the prevalence of IPV and its associated factors were gender specific. In particular, alcohol intake, family, and life satisfaction were strongly associated with both verbal and physical IPV among men and women. In addition, the prevalence of IPV victimization was significantly higher among women than among men. Moreover, older men who were dissatisfied with their family and personal life as well as heavy drinkers were more likely to be victims of physical IPV than their counterparts were. Furthermore, older women who were dissatisfied with their family and personal life as well as heavy drinkers were more likely to perpetrate physical IPV than their counterparts were. Large, prospective studies are needed to understand the etiology of these factors for the proper implementation of preventative measures to reduce IPV in South Korea.

## Conclusions

Worldwide, gender differences in IPV have been reported and, among those countries, South Korea has one of the highest prevalences of IPV. However, the factors related to IPV in Korean adults are unknown.

We found significant gender-specific differences among the factors related to IPV. In addition, the prevalences for each the type of violence perpetrated and victimized varied significantly by gender.

This is the first study to utilize nationally representative data to investigate the prevalence and risk factors of IPV in South Korea. This study measured IPV as a self-reported experience over the previous 12 months, yet further data such as sexual IPV and violence during childhood were not collected; therefore, further studies are needed.

## Abbreviations

IPV: Intimate partner violence.

## Competing interests

The authors have no conflicts of interest to disclose.

## Authors’ contributions

ML developed the research question and performed the analysis, ML and KMS drafted the manuscript and interpreted the data, ML and ECP participated in the design and planning of the study and ECP is guarantor. All authors have read and approved the final manuscript.

## Pre-publication history

The pre-publication history for this paper can be accessed here:

http://www.biomedcentral.com/1471-2458/14/415/prepub
